# Three-Dimensional Discrete Element Analysis on Tunnel Face Instability in Cobbles Using Ellipsoidal Particles

**DOI:** 10.3390/ma12203347

**Published:** 2019-10-14

**Authors:** Chao Liu, Liufeng Pan, Fei Wang, Zixin Zhang, Jie Cui, Hai Liu, Zheng Duan, Xiangying Ji

**Affiliations:** 1 School of Civil Engineering, Guangzhou University, Guangzhou 510006, China; chaoliu@gzhu.edu.cn (C.L.); 13721862830@163.com (L.P.); jcui2009@hotmail.com (J.C.); hliu@gzhu.edu.cn (H.L.); 2 Shanghai Institute of Disaster Prevention and Relief, Tongji University, Shanghai 200092, China; 3 Department of Geotechnical Engineering, College of Civil Engineering, Tongji University, Shanghai 200092, China; zxzhang@tongji.edu.cn; 4 SimuTech Lab, Boulder, CO 80309, USA; simutechlab@gmail.com; 5 Google Information Technology (Shanghai) Co., Ltd., Shanghai 200120, China; jixiangying@gmail.com

**Keywords:** shield tunneling, ellipsoids, discrete element method, face instability, micro-scale analysis

## Abstract

Soil disturbance has always been the major concern in shield tunneling activity. This paper presents the investigation on the micro-scale responses of the soils during shield tunnel excavation in sandy-cobble stratum. The code paraEllip3d is employed in discrete element method (DEM) analysis in which the soils are mimicked as an assembly of ellipsoids. Triaxial tests on the micro-scale responses of cobbles are carried out using the materials sampled from the tunnel face during construction period, and corresponding DEM simulations are performed to calibrate the micro parameters for the ellipsoids. On this basis, the face instability process during the shield tunneling in cobbles is studied using 1 g model test as well as corresponding DEM simulation. The micro-scale responses of cobbles are investigated by triaxial test as well as corresponding DEM simulations. Multiple material responses are discussed in the DEM simulations, including the stress–strain relationship, the contact distribution, and the force chain evolution in the elementary and model test. Finally, the mechanism of tunnel face instability in cobbles are discussed on the basis of aforementioned investigations.

## 1. Introduction

Shield tunneling is widely adopted for underground construction due to its gentle impact on surrounding environment and high excavation efficiency. Benefitting from the urbanization and its associated development in China, a surging number of shield tunnels with large diameters are emerging in recent decades. According to previous research [1,2], the increase of the shield tunnel diameter results in rising excavated volume, hence the decrease of tunnel face stability. For shield tunneling in clay and sand, multiple studies have been performed from the perspective of macro-scale [2,3,4] and micro-scale [5,6,7] responses of surrounding soils. However, the research on soil material responses in micro-scale during tunneling in cobbles is scarce. Different from fine-grained soil, the coarse-grained soil in tunneling is characterized by its lagging effect in face instability induced by tunneling activity. More insight in this lagging collapse frequently encountered in cobbles needs to be gained from micro-scale perspective.

Multiple approaches in macro-scale have been proposed to explore the mechanism of the face instability during tunnel excavation. For analytical approaches, different models have been developed to determine the critical support pressure at tunnel face within the framework of limit equilibrium [8,9,10,11] and limit analysis [12,13,14,15,16,17,18], respectively. For the numerical simulations based on continuum hypothesis of the materials, various studies have been carried out using Finite Element Method (FEM) [2,19,20] and Finite Difference Method (FDM) [1,21].

The macro-scale investigations aforementioned are not feasible for acquiring the insight into the micro-scale responses of the soils ahead of the collapsing tunnel face. Thus, much work has been conducted by employing the numerical approaches based on the discontinuity hypothesis of the material. The most prevalent numerical tool is the Discrete Element Method (DEM) initially developed by Cundall and Strack [22]. Several investigations on tunneling-induced soil deformation in soils have been conducted within the framework of DEM [5,7,23,24,25]. In these pioneering researches, the soils are mimicked as disks (two-dimensional analysis) or spheres (three-dimensional analysis) without considering the granularity of real soil particles. Pioneering research reveals that the rotations of disk or sphere particles extraordinarily exceed the rotations the real soil particles experience [26]. Non-spherical particles (e.g., ellipsoids) can overcome this drawback to some extent. In addition, DEM analysis has not been widely adopted in investigating soil responses in engineering problems, ascribing to the unaffordable computing cost required by the simulations. However, the development of high-performance computing (HPC) technique in recent decades has boosted the application of DEM simulation in large scale problems [27,28,29].

This paper presents a series of numerical investigation on tunnel face instability in cobble materials. The DEM code ParaEllip3d [30,31] was employed to conduct the numerical simulations with the cobble materials being mimicked by ellipsoids. The micro parameters used in the DEM model were calibrated by comparing to the triaxial tests conducted on the cobbles sampled from the excavation chamber of the shield machine employed in the Beijing Underground Cross City Railway (BJUCR) Tunnel. Thereafter, a DEM model was built to simulate the tunneling process in cobbles and the results were compared to a 1 g model test conducted at Tongji University [32]. Finally, the mechanism of the tunnel face instability in cobbles were discussed.

## 2. Discrete Element Analysis with Ellipsoids

The governing equations of the ellipsoids employed in the three-dimensional DEM simulation are identical to the spheres. For the *i*th particle, the governing equations are:

(1) $$F_{i}=m_{i}{\ddot{u}}_{i}$$

(2) $$M_{i}^{0}=I_{i}{\ddot{\theta }}_{i}$$

where ***u*** is the particle centroid displacement, ***θ*** is the spatial orientation of the ellipsoid, ***F*** is the resultant force, and ***M***^0^ is the resultant moment of the particle. **I** is the inertia moment tensor for one single ellipsoid. For the *i*th particle, its motion equation in an ellipsoid assembly can be described as:

(3) $$F_{i}=M_{i}a_{i}+C_{i}v_{i}+P_{i}$$

where ***M_i_*** denotes the generalized mass matrix of the particle, ***C_i_*** is the viscous damping matrix of the particle, ***P_i_*** stands for the generalized contact loads acted upon the *i*th particle, and ***F_i_*** is other generalized external loads on the *i*th particle including fluid drag forces and gravity. In Equation (3), ***a_i_*** and ***ν_i_*** stand for the generalized particle acceleration and velocity:

(4) $$v_{i}^{T}=[v_{x(i)},v_{y(i)},v_{z(i)},\omega_{x(i)},\omega_{y(i)},\omega_{z(i)}]$$

(5) $$a_{i}^{T}=[{\dot{v}}_{x(i)},{\dot{v}}_{y(i)},{\dot{v}}_{z(i)},{\dot{\omega }}_{x(i)},{\dot{\omega }}_{y(i)},{\dot{\omega }}_{z(i)}]$$

where *ν* is the velocity and *ω* is the angular velocity.

Equation (3) is implemented numerically using the central difference method [30]. The mid-step velocity update strategy can be expressed by assuming a mass proportional damping as follows:

(6) $$v_{n+1/2}=\frac{1-\alpha \Delta t/2}{1+\alpha \Delta t/2}v_{n-1/2}+\frac{1}{1+\alpha \Delta t/2}\Delta tM^{-1}\left[F_{n}-P_{n}\right]$$

where *n* is the *n*th step, Δ*t* denotes the time-step. *α* is the proportionality parameter between mass and damping (***C*** = *α**M***, ***C*** and ***M*** denote particle damping and mass, respectively). Two kinds of damping, i.e., background damping and contact damping, are usually employed in DEM simulation [31]. For physical dynamic simulation on particles, the contact damping is usually adopted without employing background damping for the whole granular system. The contact damping force in the normal direction between two contacting particles can be expressed as:

(7) $$F_{d}=c_{r}v_{r}$$

where ***ν_r_*** is the normal relative velocity vector of the centers in two contacting particles. Commonly, the normal contact damping coefficient *c_r_* is treated as a fraction of the critical damping coefficient *C_r_* of the system composed of two rigid bodies:

(8) $$C_{r}=2\sqrt{\frac{m_{1}m_{2}k_{n}}{m_{1}+m_{2}}}$$

(9) $$c_{r}=\xi C_{r}$$

where *m*_1_ and *m*_2_ are the masses of the two particles and *k_n_* denotes the stiffness of the spring connecting the two particles. *ξ* is the damping ratio.

The Hertz–Mindlin model is used in the DEM simulation in the paper. For normal contact force between particles, Hertz contact model is implemented in the code to mimic an elastic contact behavior between two particles. For tangential contact force between particles, Mindlin contact model is employed, considering the initial state of loading and the load history in both normal and tangential directions. The details of Hertz–Mindlin model can be found in various existing studies [26,31], hence no presence here in the paper.

Inherently, the contact detection between ellipsoids is more difficult than that between spheres, ascribing to the complexity of the ellipsoidal contact geometry. The pioneering work carried out by Lin and Ng [33] proposed two different algorithms for the contact detection between two ellipsoids. One is based on a common normal concept and the other on a geometric potential concept. For the algorithm based on the common normal concept, the computational cost is extremely large to obtain a converging solution, although the algorithm itself meets better the substantial definition of a contact [34]. Therefore, the algorithm employed in this study is on the basis of the geometric potential concept [31]. The details of the algorithm used herein can be found in the previous research [31], hence not being presented in this paper.

## 3. Calibration of the Micro Parameters

### 3.1. Triaxial Test

**Figure 1:**
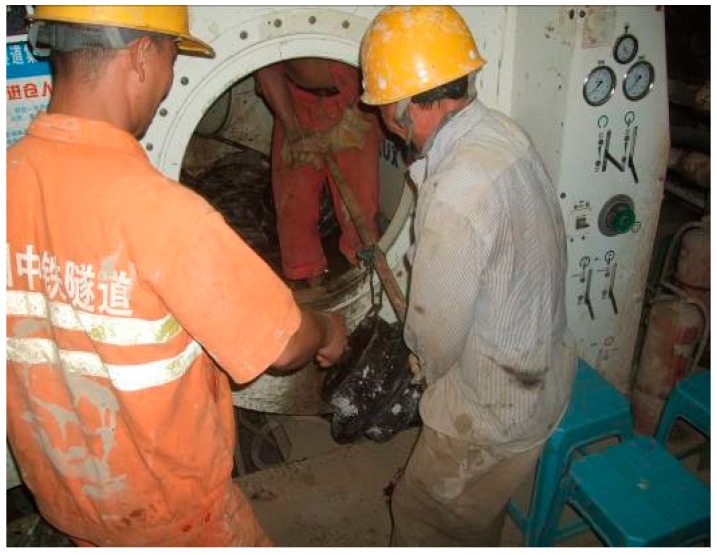
Soil sampling at the tunnel face.

**Figure 2:**
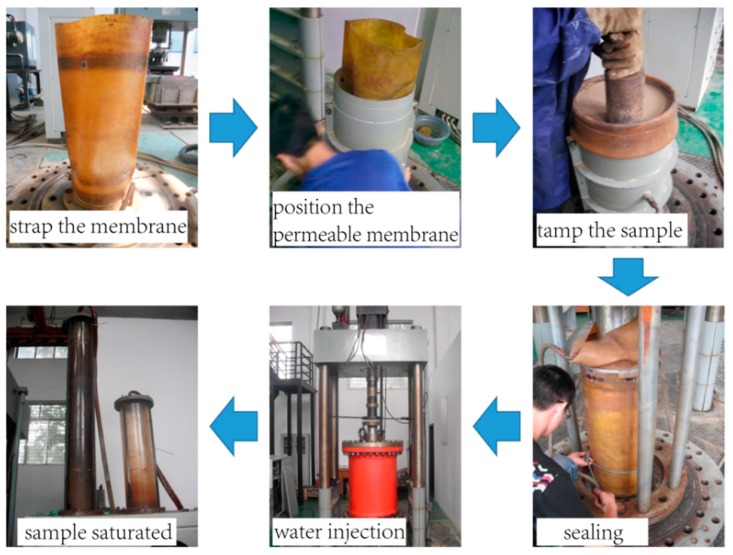
The triaxial test procedure.

Prior to the discrete element modeling for the tunnel face instability, a series of triaxial tests were performed on one sample at Wuhan Institute of Geotechnical Engineering, Chinese Academy of Sciences. The cobbles used in the tests were sampled from the BJUCR Tunnel project. In the halt of the shield machine during the tunneling process, the in-situ soils at the excavation surface was collected by the pressure opening technology (as shown in Figure 1), and then transported to the lab at Wuhan. In the preparation of the sample, the cobbles were weighted according to the gradation ratio after removing the oversize soil mass, and the mixed soil samples were fully mixed to generate the samples. The density of the specimen in this test was 22 kN/m^3^. The size of the specimen was ∅ 300 mm × 600 mm (as shown in Figure 2). The specimen was filled with 4 layers with each layer 150 mm in height. Each layer was tamped from loose to 150 mm with the measurement error of the last layer less than ±2 mm. The specimen was loaded in multiple stages at 200 kPa, 400 kPa, and 600 kPa. Figure 2 illustrates the whole procedure of the triaxial test.

**Figure 3:**
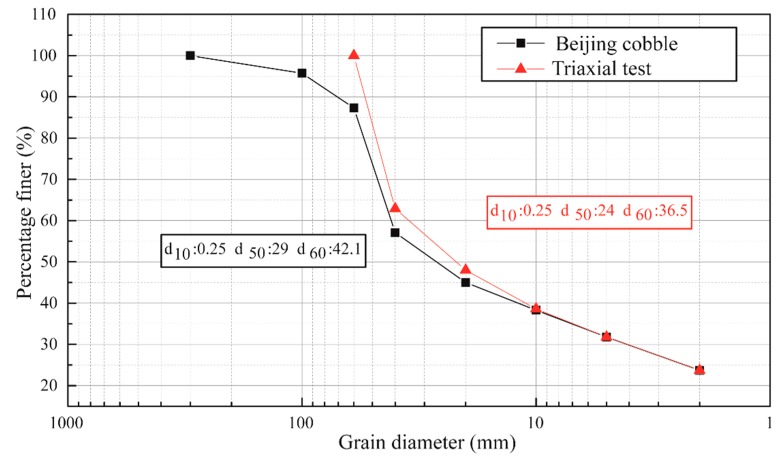
Grading curve of the in-situ soils and the sample used in the triaxial test (d is the maximum diameter of one single particle).

Figure 3 shows the grading curve of the in-situ soil at Beijing and the sample soil used in the triaxial test. As can be observed from the comparison in Figure 3, the grading curve of the soil sample used in the triaxial test was analogous to that of the in-situ soil at Beijing, except that in the test the cobbles larger than 60 mm in diameter were eliminated from the sample to make the triaxial test easy to perform.

### 3.2. Preparation of Discrete Element Samples

**Figure 4:**
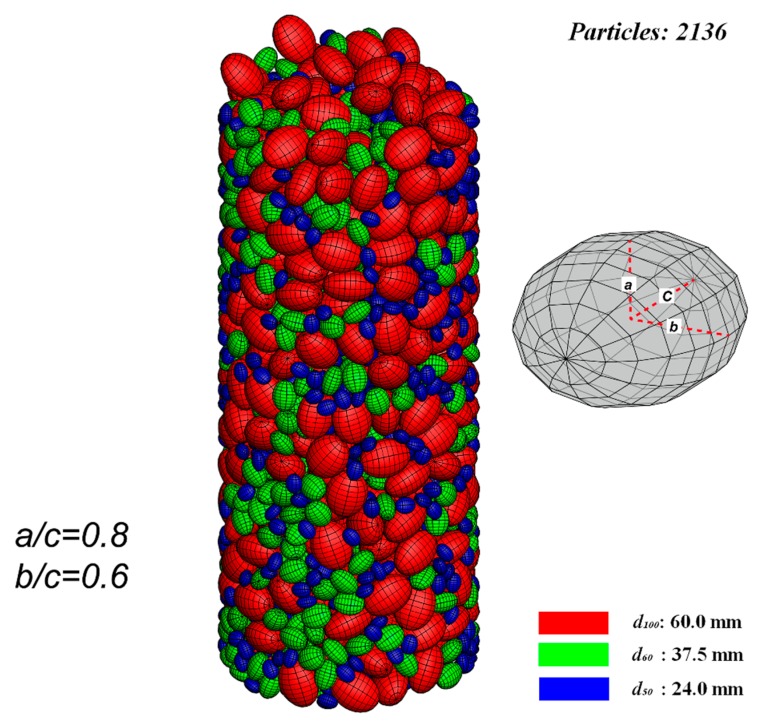
The cobble sample after deposition.

**Figure 5:**
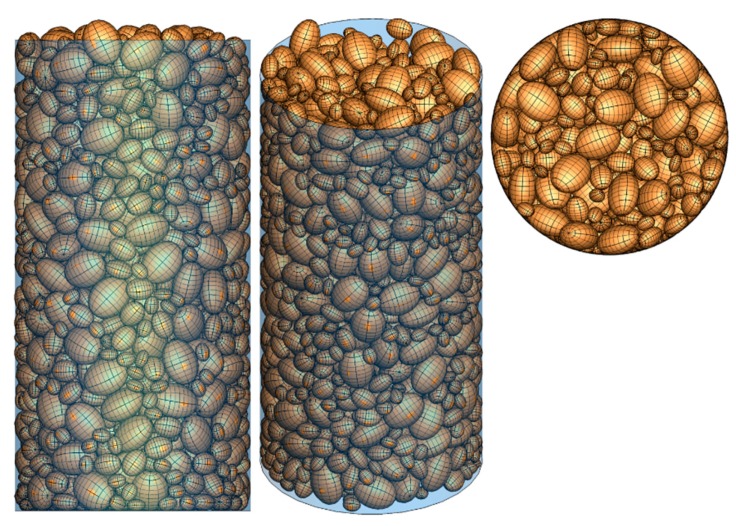
Removal of the particles outside of the specimen.

**Figure 6:**
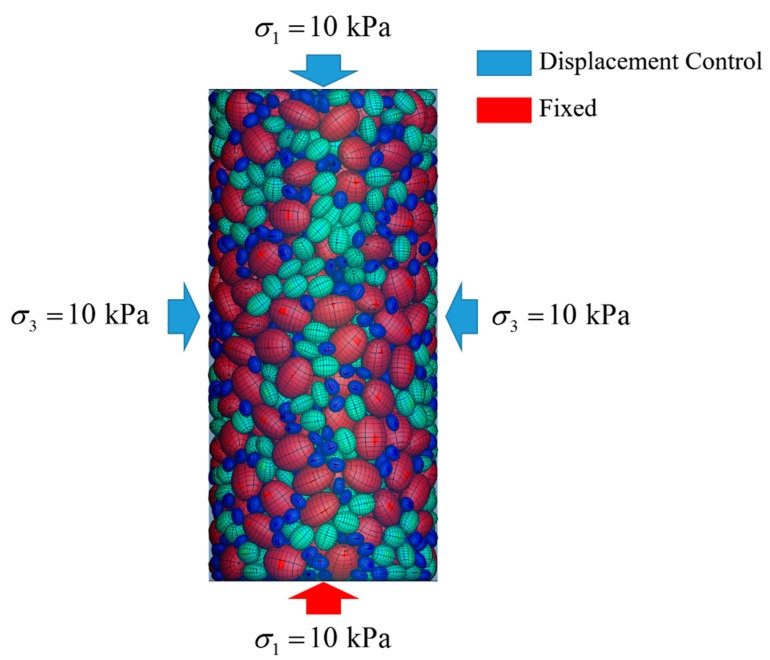
Boundary condition and loading of the triaxial test.

Generally, two sampling techniques in discrete element modeling are prevalently adopted [35]: (1) Dynamic technique in which the sample is generated by particle deposition; and (2) constructive algorithms in which the sample is created based on particle geometries. In this study, the preparation of the discrete element model was performed by particle deposition in the following steps:

(1) A number of particles are randomly generated, floating over the bottom of the container. In order to avoid the decrease of computational efficiency induced by excessive small particles, three particle sizes, i.e., d_100_ = 60 mm (d_100_: the grain diameter at 100% passing), d_60_ = 36.5 mm (d_60_: the grain diameter at 60% passing), and d_50_ = 24 mm (d_50_: the grain diameter at 50% passing) are selected in the simulation to generate particles. According to the sieve analysis performed prior to the triaxial compression test, the particles in the soil sample are characterized by *a*/*c* = 0.6 and *b*/*c* = 0.8, where *a*, *b*, and *c* denote the half the length of three principal axes of each ellipsoid with the relationship of *a* < *b* < *c* (as depicted in Figure 4).
(2) As illustrated in Figure 4, a total number of 2136 particles are deposited finally to form the cobble sample used in following DEM simulation on triaxial tests;
(3) The sample was deposited with a final height slightly greater than the height of the specimen of the triaxial test (i.e., 600 mm), and the particles outside of the specimen were deleted from the model (as shown in Figure 5);
(4) A small pressure (10 kPa herein) was applied to the sample to make the particles fully contacted with the boundaries. Displacement boundaries (servo control) were applied to the top of the specimen. The boundaries were assumed to be rigid. It should be pointed out that the bottom of the specimen was fixed in the vertical direction according to the test prototype (as shown in Figure 6);
(5) A confining pressure of 200 kPa was applied to the specimen for the isotropic compaction prior to triaxial loading;
(6) The triaxial compression test was performed by moving the top boundary downward. The confining pressure *σ*_3_ is maintained at 200 kPa by adjusting the diameter of the cylindrical boundary at each time-step.

During the preparation of the sample, the coefficient of friction between particles is 0.5, and the coefficient of friction between the particles and boundary is 0.5.

### 3.3. Microscopic Material Parameters

The parameters of particle materials in the discrete element simulation include Young’s Modulus, friction coefficient, shear modulus, damping coefficient, and particle density. In this test, it is necessary to calibrate the friction coefficient between particles and that between particles and boundaries.

The microscopic parameters involved in the modeling cases are shown in Table 1. In the calibration stage, a total of 8 different cases were calculated to obtain the particle parameters that were consistent with the results of the laboratory test mentioned above. It should be pointed out that, in order to make the calculation results accord with the actual situation in the triaxial tests, a local damping was employed on the particles instead of global damping. This could reduce the particle vibration caused by the elastic assumption in particle contacts.

**Figure 7:**
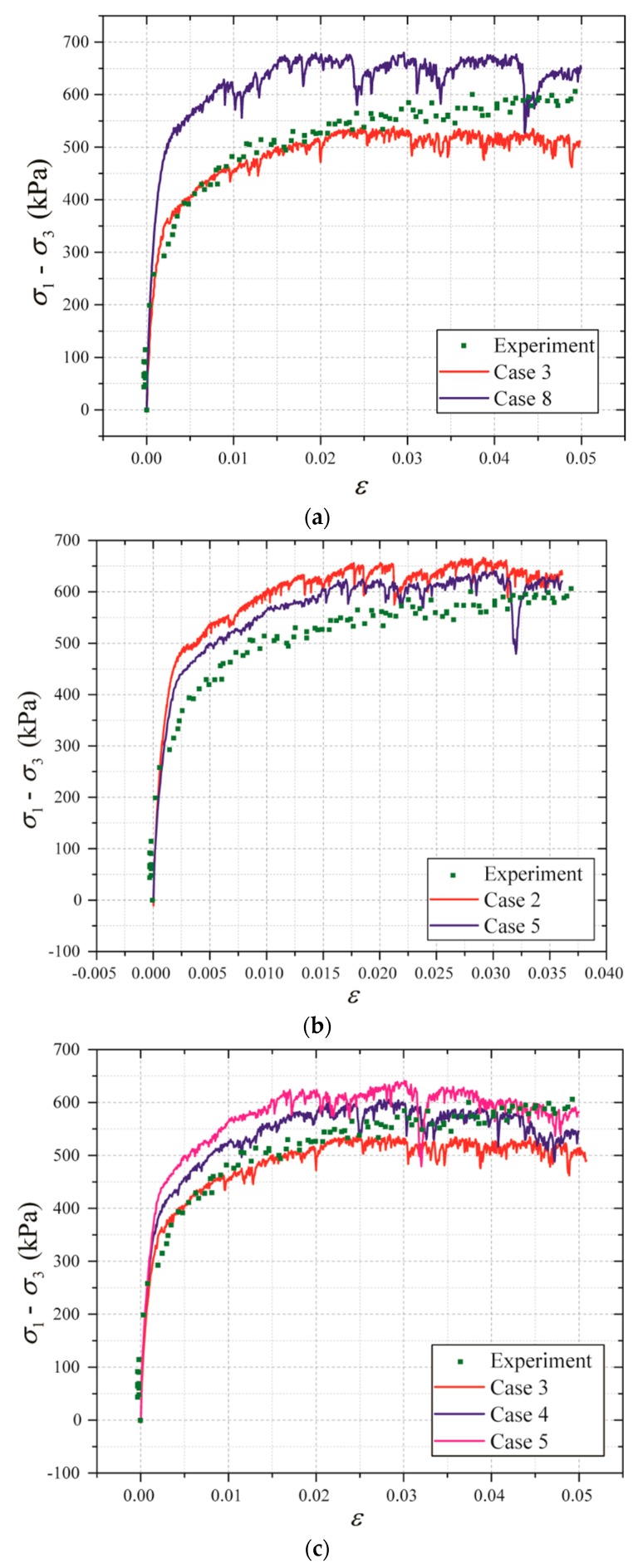

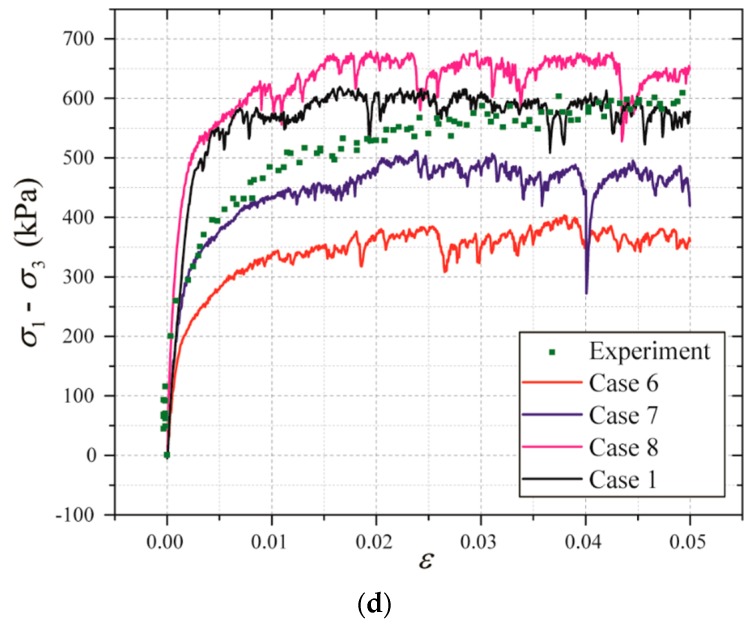
Comparison between DEM results and triaxial test data (confining pressure: 200 kPa): (**a**) the deviatoric stress–strain curves in Case 3 and 8; (**b**) the deviatoric stress–strain curves in Case 2 and 5; (**c**) the deviatoric stress–strain curves in Case 3, 4, and 5; and (**d**) the deviatoric stress–strain curves in Case 1, 6, 7, and 8.

Figure 7 show the comparison between DEM results and triaxial test data at the confining pressure of 200 kPa. The calibration results are listed in Table 2. According to the calibration results, the micro-parameters of Case 4 can be employed in tunneling simulation using DEM.

### 3.4. Evolution of the Sample Micro-Structure

#### 3.4.1. Gravity Deposition Stage

**Figure 8:**
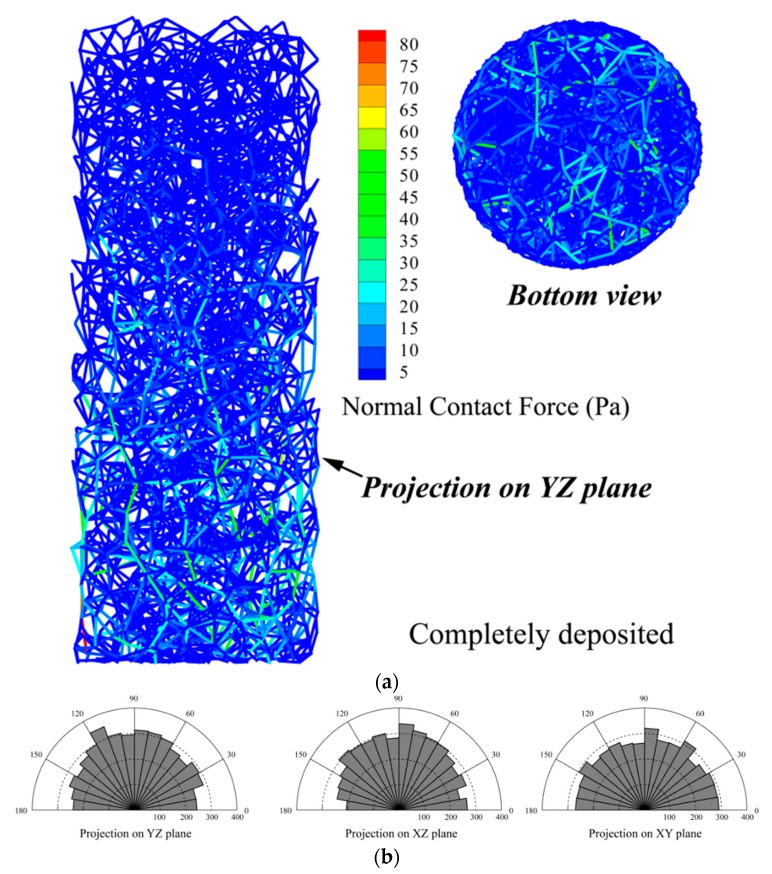
Micro-structure of the cobble sample after deposition: (**a**) distribution of the force-chain; and (**b**) rose plot of the contact direction in particles.

Figure 8 shows the distribution of force chain and the contact between particles in the samples after the deposition under gravity. As illustrated in Figure 8a, the contact force is larger at the bottom than other location in the sample, ascribing to the gravity, and the strong force chain distribution is relatively uniform from the bottom view. In Figure 8b, the circumferential axis indicates the direction of the contact normal in the polar coordinate system (at an interval of 10°), and the radial axis represents the number of contacts within the direction interval. It can be seen that the normal direction distribution of the particle contact is relatively uniform. However, in the XZ plane of Figure 8b, the number of contacts in the normal direction from 170° to 180° is relatively small, since the sample has not been flattened on the top after the particle deposition. The projection on the XY plane (i.e., the top-view plane) in the contact normal direction is relatively uniform, indicating the sample is well deposited.

#### 3.4.2. Isotropic Compression under a Confining Pressure of 200 kPa

**Figure 9:**
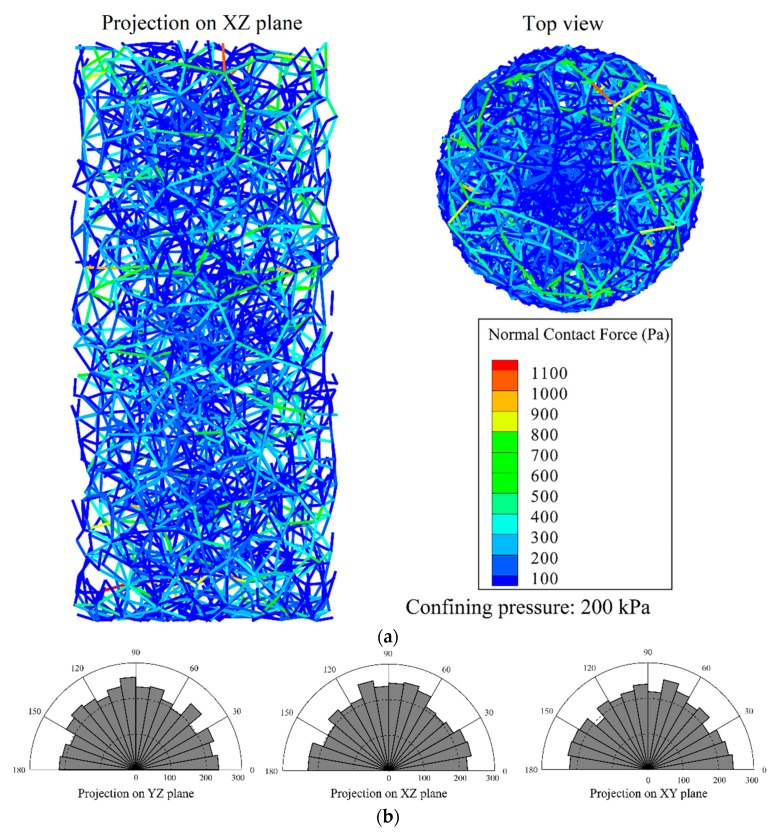
Micro-structure of the cobble sample during isotropic compression (confining pressure: 200 kPa): (**a**) distribution of the force-chain; and (**b**) rose plot of the contact direction in particles.

Figure 9 shows the sample micro-structure after the isotropic compression under a confining pressure of 200 kPa. It can be seen from the distribution of the force chain that after the isotropic compression, the strong contact of the particles inside the sample is more than that after the gravity deposition with much greater contact force. In addition, most strong force chains are distributed circumferentially at the periphery of the specimen from the perspective of top-view. As can be observed from the top view, the number of strong force chains at the center of the sample is relatively small. This is because the top particle is looser than those at the bottom, resulting in a high void ratio at the sample top. It can be seen from the rose plot of the contact direction that the contact direction distribution at this time is relatively uniform.

#### 3.4.3. Triaxial Compression (Confining Pressure 200 kPa)

**Figure 10:**
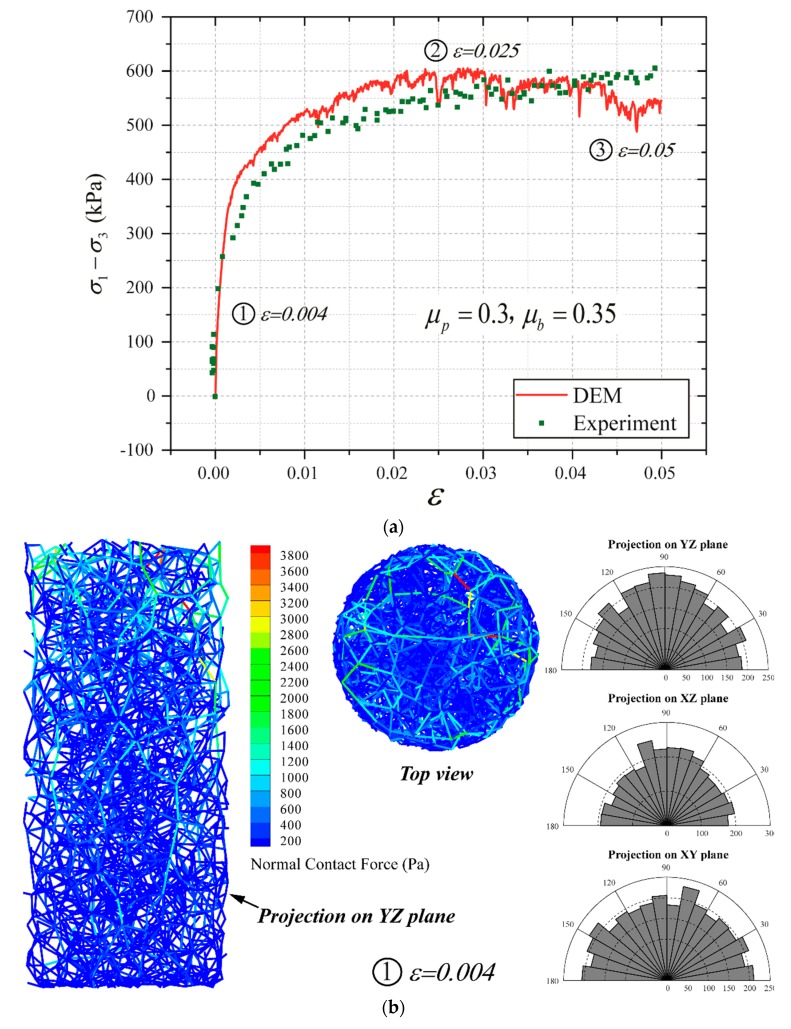

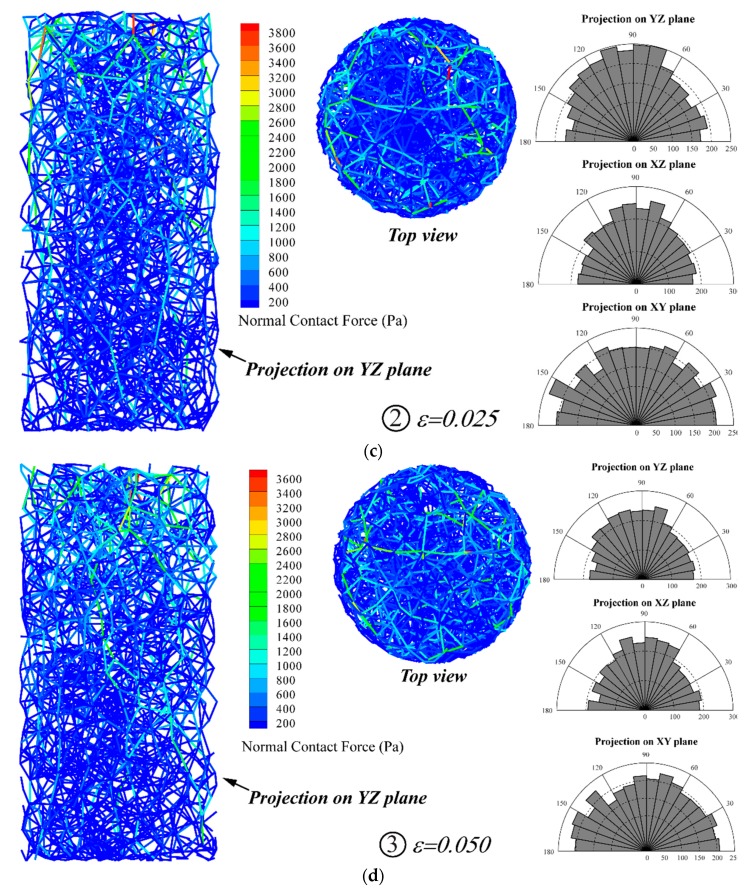
Micro-structure evolution of the cobble sample during triaxial compression (confining pressure: 200 kPa): (**a**) deviatoric stress–strain curve; (**b**) the sample micro-structure at *ε* = 0.004; (**c**) the sample micro-structure at *ε* = 0.025; and (**d**) the sample micro-structure at *ε* = 0.050.

Figure 10 shows the variation of the sample micro-structure at different stages during the triaxial compression loading. As shown in Figure 10a, three loading stages are selected for the analyses: (1) *ε* = 0.004, which represents the initial elastic phase; (2) *ε* = 0.025, which corresponds to the peak value of the partial stress; and (3) *ε* = 0.050, representing the completion of the triaxial compression after which the sample shows strain softening to a certain extent.

Figure 10b illustrates the distribution of the force chain and the particle contact rose diagram of the sample at the loading state of *ε* = 0.004. Comparing with Figure 9a, it can be concluded that the contact force and the number of the strong force chain inside the sample gradually increase as the compression progresses, indicating that the internal pressure is increasingly borne by the strong contact force chain. At the same time, it can be observed seen that the strong contact in horizontal direction gradually decreases. As can be seen from the rose plot on the distribution of the contact direction, progressive contacts emerge in the vertical direction. Figure 10c shows the changes in the sample micro-structure when the deviatoric stress reaches the peak value. Comparing with Figure 7b, it can be found that the contact direction between particles progressively develops along the vertical direction, indicating that the stress is more and more concentrated in the vertically distributed strong force chains. For a single strong force chain in stage, it can be found that no obvious substitution of the strong force chain occurs, indicating that no significant shear failure emerges inside the sample at this time and the shear force on the sample is still borne by the strong force chain formed during the elastic stage aforementioned.

Figure 10d presents the sample micro-structure variation at the end of the test (i.e., *ε* = 0.050). The maximum contact force exhibited in Figure 10d decreases compared with the previous stage (i.e., *ε* = 0.025), which exactly corresponds to the strain softening phenomenon on the macroscopic scale. However, comparing with the previous loading stage, there is no significant change at this loading stage in the distribution of the strong force chain and the contact normal direction (see the rose plots in Figure 10d), indicating that the softening of the sample is not obvious at this time. This corresponds to the observation from the deviatoric stress–strain curve of the sample (shown in Figure 10a).

## 4. Discrete Element Modeling for 1 g Model Test of Tunnel Face Instability

### 4.1. Revisit of the 1 g Model Test

**Figure 11:**
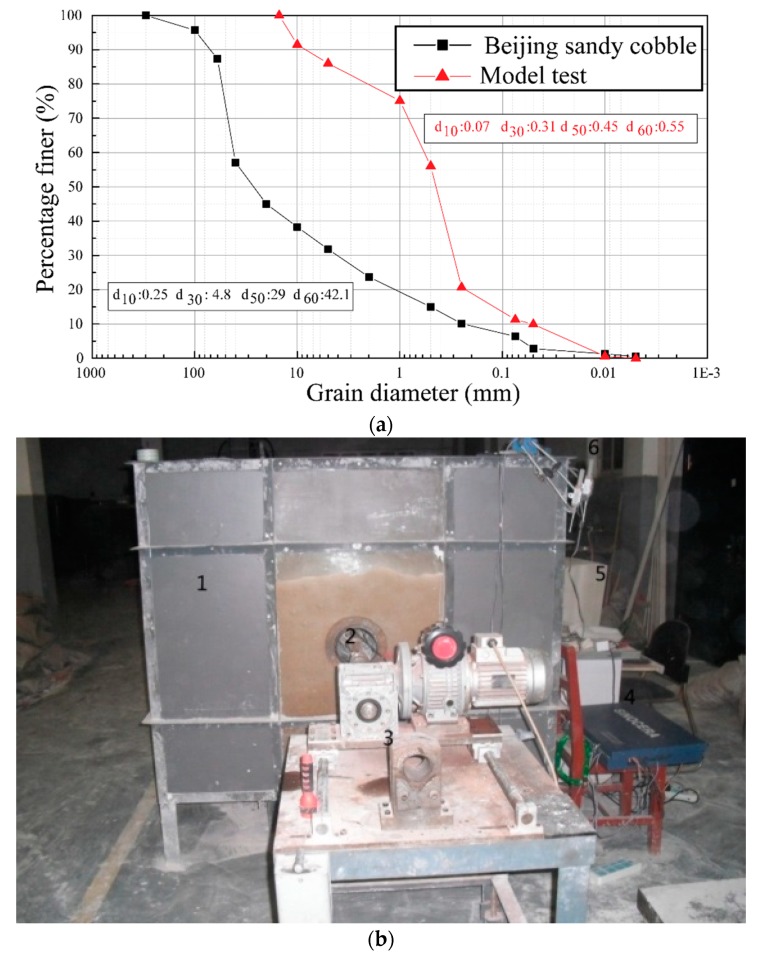
Overview of the 1 g model test: (**a**) Gradation of the soil material used in the 1 g model test (d stands for the maximum diameter of one single particle); and (**b**) prototype of the model [32].

In order to investigate the tunnel face instability in cobble stratum, a previous research [32] has conducted a series of 1g model tests on face collapse during tunneling in cobbles. The materials used in the model test was selected according to the sandy cobble stratum encountered in the BJUCR Tunnel project (as shown in Figure 11a) [32]. The gradation used in the model test is configured according to the principle of dimensional homogeneity. A new earth pressure balance shield test system was employed in the test, which consisted of a model shield system, a model tank, a sensor measurement system, and a shield power system (see Figure 11b). Dimension of the soil tank was 1500 mm × 1200 mm × 1300 mm (length × width × height). The model shield is made of aluminum alloy with a length of 260 mm, an outer diameter of 164 mm, an inner diameter of 160 mm, and a shield shell thickness of 4 mm. In the model test, the effects of shield driven, soil cutting, and ground surcharge on tunnel face instability were considered.

### 4.2. DEM Simulation on the 1 g Model Test

#### 4.2.1. Parallelization of the Computation

In order to improve the computational efficiency, it is necessary to parallelize the calculated objects. Simultaneously, in order to perform parallel computations on high-performance computing (HPC) clusters, it is necessary to use the MPI-based parallel computing method to discretize the computational domain, so that the whole model can be processed on multiple nodes/processors in a distributed way. In this study, a hybrid MPI-OpenMP approach is employed for the DEM simulations on the 1 g model test, since the computational workload is extremely high (up to more than 60,000 ellipsoids) and HPC cluster is left to be the only choice. Details of the hybrid OpenMPI-OpenMP approach can be found at Yan and Regueiro [27].

#### 4.2.2. Discrete Element Modeling for the Tunnel Face Instability

**Figure 12:**
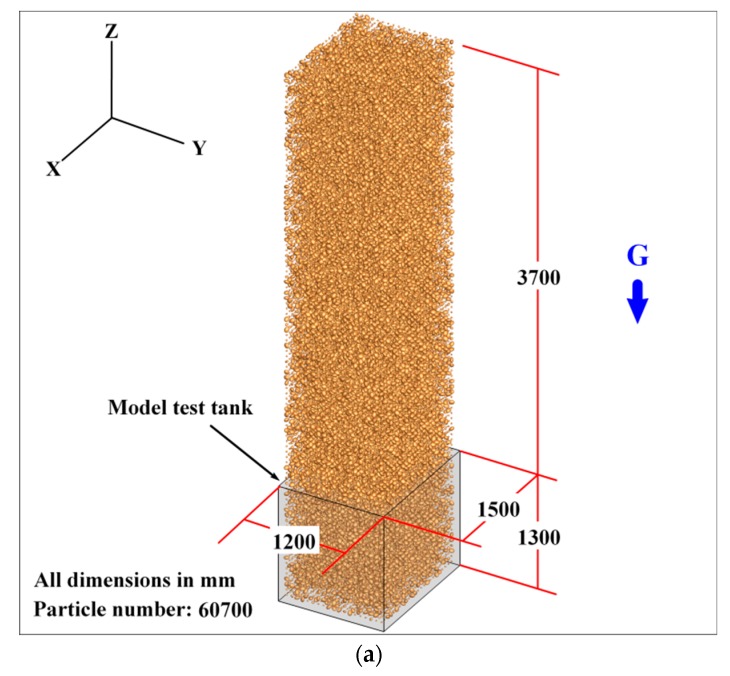

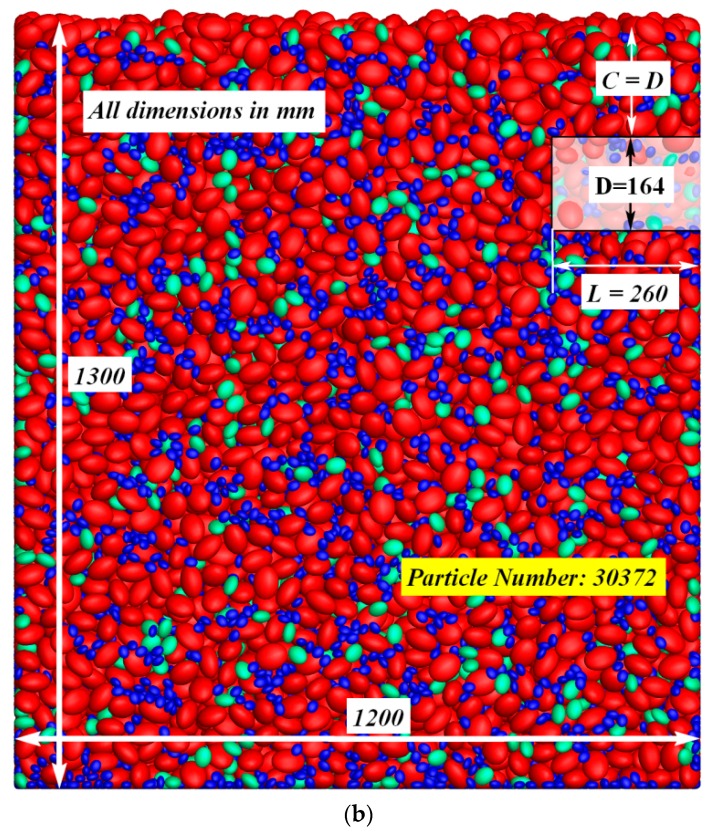
Overview of the discrete element model for the 1 g model test (all dimensions in mm): (**a**) Model tank and particles prior to deposition; and (**b**) tunnel excavation region inside the model tank.

Before the tunnel excavation simulation, it is necessary to generate the samples required for the discrete element modeling. However, if the actual particle size in the model test is adopted for DEM simulation, the number of particles involved will be in the hundreds of millions which is obviously unrealistic for the computational ability of current HPC clusters. Therefore, in order to conduct a qualitative analysis of the tunnel face instability, the actual particle gradation of the Beijing cobble stratum is used in the discrete element model. The specimen is generated by gravity deposition in the DEM simulation (see Figure 12). A total number of 60,700 particles are generated in the model.

In order to reduce the computational cost, the model is simplified to be symmetric about the vertical plane where the tunnel axis is located, and only half of the particles (30,372 in total) are considered in tunneling simulation. In addition, this treatment makes it convenient to observe the particle movement during the tunnel face instability. After the particle deposition, the particles within the excavation area will be deleted to mimic the tunnel effect (as shown in the shadowed area in Figure 12). The micro-parameters in the DEM model, i.e., the friction coefficient between two particles, as well as that between particle and boundary, are configured according Case 4 in Section 3.

The gravity deposition process continues until the maximum particle velocity is less than the given value (0.01 m/s in this simulation). This process is necessary for making the particles fully in contact with the tunnel boundary after the removal of the particles inside the tunneling region in stage 2;

The progressive tunnel face instability is simulated under 1 g gravity condition. The tunnel excavation is simulated by deleting particles that are in contact with the boundary of the excavation face. In order to improve the calculation efficiency and avoid the calibration of the tunneling variables (e.g., tunneling speed and face supporting pressure) [5], the shield driven process is not considered in this simulation.

**Figure 13:**
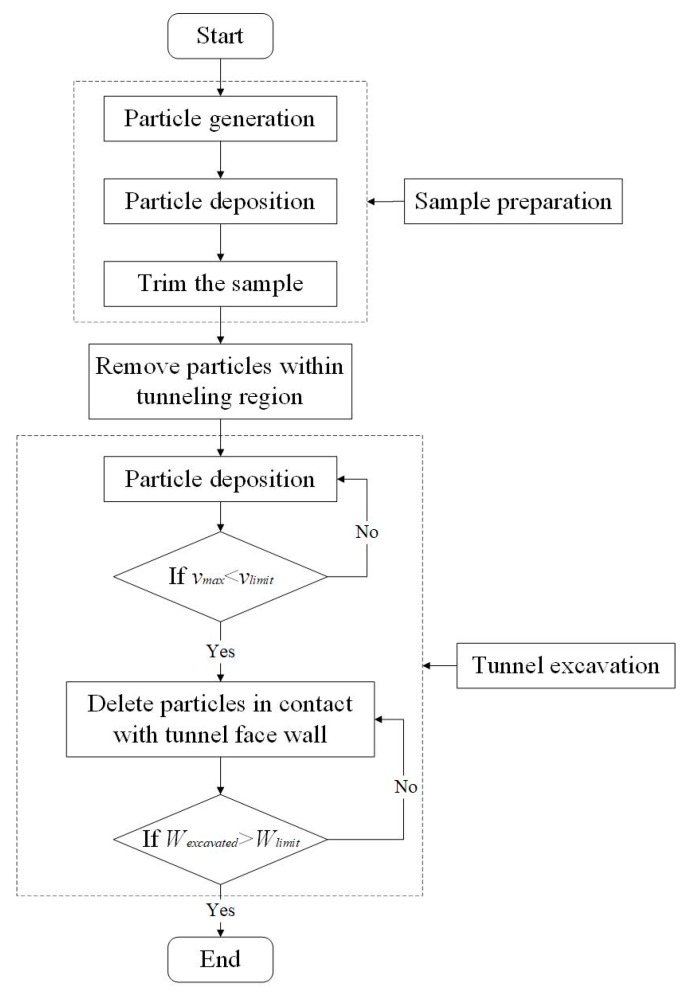
Flow chart for the computational process (*v_max_*: the maximum velocity of the particles; *v_limit_*: velocity limit which is close to zero; *W_excavated_*: total weight of the removed particles; and *W_limit_*: weight limit for the deposition calculation in tunneling simulation).

Two cover-diameter ratios are considered in the DEM simulation, i.e., *C*/*D* = 1.0 and *C*/*D* = 2.0 (*C* and *D* are the cover and diameter of the tunnel, respectively). The entire calculation process is performed on an HPC cluster using 1200 processes in parallel, with one single case (tunneling in 1.5 s) calculated for approximately 72 h. The whole simulation procedure is summarized as follows and illustrated in Figure 13.

### 4.3. Results and Discussion

#### 4.3.1. *C*/*D* = 1.0

**Figure 14:**
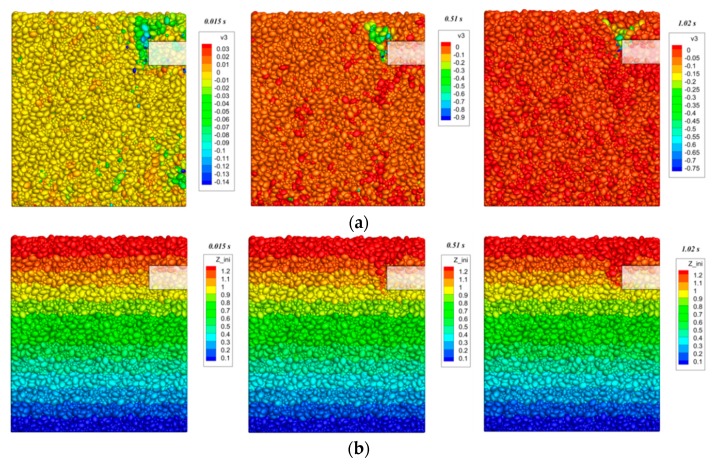
The collapse of tunnel face (*C*/*D* = 1.0): (**a**) Speed distribution of particles (vertical direction, unit: m/s); and (**b**) displacement distribution of particles (vertical direction, unit: m).

Figure 14 shows the development of particle velocity and displacement during tunnel excavation under *C*/*D* = 1.0. It can be seen from Figure 14a that as the excavation progresses, the particles gradually move toward the excavation surface, and at the same time the velocity distribution exhibits a typical basin failure pattern of the tunnel face. It can be seen from the velocity development trend that the particles behind rather than those ahead of the excavation face experience velocity first.

**Figure 15:**
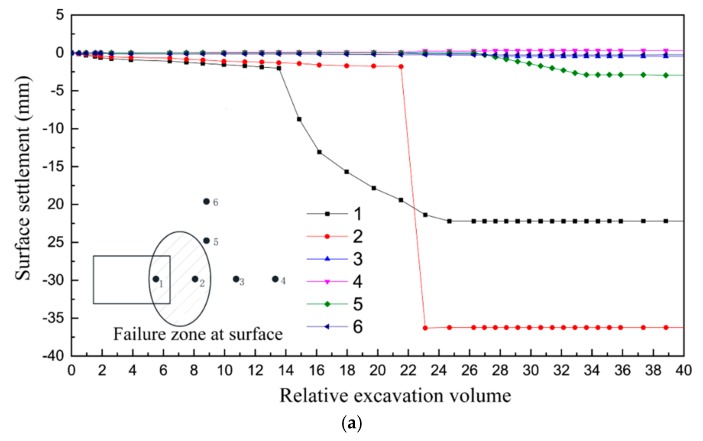

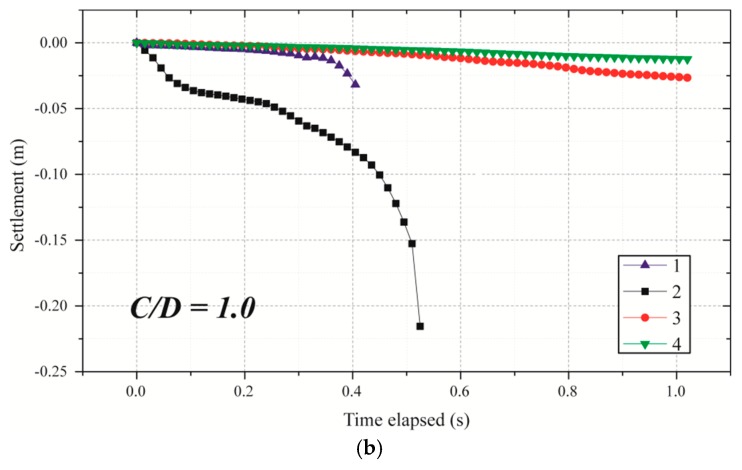
Evolution of the surface settlement during tunnel face instability (*C*/*D* = 1.0): (**a**) results of the 1 g model test [36]; and (**b**) results of discrete element method (DEM) simulation.

Figure 15 illustrates the surface settlement development obtained from the DEM simulation and the 1 g model test, respectively. It can be found that, for monitoring point 2, the sudden settling occurred in the numerical simulation at 0.5 s, indicating that the global collapse had occurred at this moment (the surface particles collapsed to the excavation face). For the soils at measuring points 3 and 4, no instability phenomenon occurred either in numerical simulation or model test. Comparing with measuring point 3, the instability is more likely to occur at point 1, which is revealed in both numerical simulation and model test.

#### 4.3.2. *C*/*D* = 2.0

**Figure 16:**
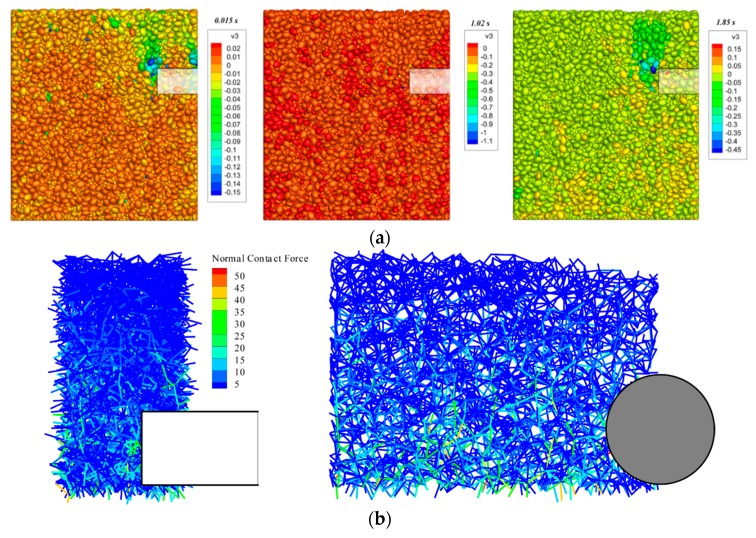
Micro-structure evolution during tunnel face collapse under *C*/*D* = 2.0: (**a**) Particle speed distribution; and (**b**) distribution of the force chain while the tunnel face is collapsing.

Figure 16 represents the development of soil particle velocity during excavation in the case of *C*/*D* = 2.0. As can be observed from the Figure 16a, the velocity of the particles near the vertical plane where the tunnel axis located is significantly smaller than that of *C*/*D* = 1.0 (see Figure 14a), indicating that an arching effect emerges in the model while *C*/*D* = 2.0. Figure 16b shows the distribution of particle force chains around the excavation area at 2.45 s. It can be seen from the figure that although there is no obvious arching effect in the soil due to the development of surface subsidence, the strong force chain locates in the vicinity of the excavation zone. In addition, it can be observed that there are a certain number of strong chains distributed around the tunnel, indicating that the soil-arching is forming gradually at this stage (i.e., collapsing time equals to 2.45 s).

## 5. Conclusions

The paper presents an investigation on the micro-scale responses of cobbles during tunnel face instability. A series of DEM simulations using three-dimensional ellipsoids were performed on the triaxial compression tests of Beijing cobbles and the 1 g model test on BJUCR Tunnel project in Beijing. The micro-parameters were calibrated by the comparison between discrete element modeling and triaxial compression test conducted in the lab, and the evolution of micro-structure was investigated simultaneously. Subsequently, the 1 g model test was modeled using DEM and the micro-responses of the cobbles was investigated at the same time.

The following conclusions can be drawn:

1. It can be found from the laboratory triaxial compression test and discrete element modeling that the cobble sample does not show obvious strain softening property under the confining pressures of 200 kPa, 400 kPa, and 600 kPa; The cobble sample in the discrete element simulation shows softening behavior to a certain degree, ascribing to the removal of small particles in the discrete element model. The macro properties of the cobble sample (e.g., material softening) can be investigated microscopically via the variation of the force chain distribution;
2. The tunnel excavation model test was simulated using the calibrated friction parameters, considering the two cover-diameter ratios (i.e., *C*/*D* = 1.0 and *C*/*D* = 2.0). A basin-like failure pattern can be observed for the tunnel face instability while *C*/*D* = 1.0, and a chimney-like failure pattern for the case of *C*/*D* = 2.0. For the case of *C*/*D* = 2.0, although the overall instability of the excavation surface did not occur during the calculation time, the surface settlement did not stabilize within 2.45 s. It can also be found from the force chain distribution that the arching effect of the cobble is forming gradually during the tunnel face instability while *C*/*D* = 2.0.
3. The DEM simulation using ellipsoidal particles can obtain higher shear strength than spherical particles, but the computational efficiency is extremely low. This is not acceptable for the analysis of large-scale problems. However, with the development of computational power, the analysis of engineering problems will become feasible in the future.

## Figures and Tables

**Table 1:** Microscopic of the particle material.

Cases	*E* (Pa)	*ν*	*μ_p_*	*μ_b_*	*ξ*
Case 1	2.0 × 10^10^	0.25	0.5	0.5	0.03
Case 2	2.0 × 10^10^	0.25	0.4	0.4	0.03
Case 3	2.0 × 10^10^	0.25	0.3	0.3	0.03
Case 4	2.0 × 10^10^	0.25	0.3	0.35	0.03
Case 5	2.0 × 10^10^	0.25	0.3	0.4	0.03
Case 6	2.0 × 10^10^	0.25	0.5	0	0.03
Case 7	2.0 × 10^10^	0.25	0.5	0.1	0.03
Case 8	2.0 × 10^10^	0.25	0.5	0.3	0.03

*E*: Elastic modulus; *ν*: Poisson’s ratio; *μ_p_*: The friction coefficient between particles; *μ_b_*: The friction coefficient between particles and rigid boundary; *ξ*: Contact damping ratio.

**Table 2:** Microscopic of the particle material.

Cases	*E_sample_* (MPa)	*E*_50_ (MPa)	Strength (kPa)
Experiment	649.5	201.6	609.6
Case 1	382.3	211.3	619.4
Case 2	660.1	355.9	670.2
Case 3	468.4	237.4	541.6
Case 4	755.9	268.3	606.7
Case 5	532.8	300.0	643.2
Case 6	215.9	123.1	404.8
Case 7	337.5	183.1	514.4
Case 8	993.1	391.9	680.9
